# The Transcriptomic Evidence on the Role of Abdominal Visceral vs. Subcutaneous Adipose Tissue in the Pathophysiology of Diabetes in Asian Indians Indicates the Involvement of Both

**DOI:** 10.3390/biom10091230

**Published:** 2020-08-24

**Authors:** Anshul Kumar, Pradeep Tiwari, Aditya Saxena, Naincy Purwar, Nitin Wahi, Balram Sharma, Sandeep Kumar Mathur

**Affiliations:** 1 Department of Endocrinology, Sawai Man Singh Medical College and Hospital, Jaipur 302004, India; anshul.singh2910@gmail.com (A.K.); tiwari.pradeep30@gmail.com (P.T.); naincypurwar@yahoo.co.in (N.P.); drbalramendo@gmail.com (B.S.); 2 Department of Chemistry, School of Basic Sciences, Manipal University Jaipur, Jaipur 303007, India; 3 Department of Biotechnology and Bioinformatics, Birla Institute of Scientific Research (BISR), Jaipur 302001, India; 4 Department of Biotechnology, Institute of Applied Sciences and Humanities, GLA University, Mathura 281406, India; aditya.235@gmail.com; 5 Department of Bioinformatics, Pathfinder Research and Training Foundation, Gr. Noida 201308, India; wahink@gmail.com

**Keywords:** adipocyte, Asian-Indians, diabetes, transcriptome

## Abstract

The roles of abdominal visceral (VAT) and subcutaneous adipose tissue (SAT) in the molecular pathogenesis type-2 diabetics (T2D) among Asian Indians showing a “thin fat” phenotype largely remains obscure. In this study, we generated transcription profiles in biopsies of these adipose depots obtained during surgery in 19 diabetics (M: F ratio, 8:11) and 16 (M: F ratio 5:11) age- and BMI-matched non-diabetics. Gene set enrichment analysis (GSEA) was used for comparing transcription profile and showed that 19 gene sets, enriching inflammation and immune system-related pathways, were upregulated in diabetics with F.D.R. <25% and >25%, respectively, in VAT and SAT. Moreover, 13 out of the 19 significantly enriched pathways in VAT were among the top 20 pathways in SAT. On comparison of VAT vs. SAT among diabetics, none of the gene sets were found significant at F.D.R. <25%. The Weighted Gene Correlation Analysis (WGCNA) analysis of the correlation between measures of average gene expression and overall connectivity between VAT and SAT was significantly positive. Several modules of co-expressed genes in both the depots showed a bidirectional correlation with various diabetes-related intermediate phenotypic traits. They enriched several diabetes pathogenicity marker pathways, such as inflammation, adipogenesis, etc. It is concluded that, in Asian Indians, diabetes pathology inflicts similar molecular alternations in VAT and SAT, which are more intense in the former. Both adipose depots possibly play a role in the pathophysiology of T2D, and whether it is protective or pathogenic also depends on the nature of modules of co-expressed genes contained in them.

## 1. Introduction

Diabetes is an enigma known to man since ancient times, but, even today, its precise causation and pathogenesis eludes us. It is known that the pathogenesis of diabetes is linked to obesity and, in the case of Asian Indians, specifically to visceral adiposity [1,2,3,4]. As compared to other ethnicities, they show relatively higher insulin resistance and have a higher risk of developing metabolic syndrome despite lower body mass index (BMI). This paradox is attributed to their typical “thin, fat phenotype” characterized by not only an increased overall body fat content but also a peculiar fat distribution pattern characterized by excess intra-abdominal fat deposition [5,6,7]. This unusual fat distribution pattern is hypothesized to occur due to “the nutrient overflow” [8,9,10]. According to this, a smaller subcutaneous fat compartment owing to its limited capacity to store excess calories leads to the accumulation of excess fat in the intra-abdominal compartment comprising of the omentum and ectopic fat deposition in the liver, muscle, and other organs. Several clinical and epidemiological studies have found an association between visceral fat deposition and insulin resistance and metabolic syndrome in this population [11,12,13,14], while the abdominal subcutaneous adipose tissue is usually considered to be protective [15,16]. In addition, it has been observed that, despite having lower body weights, even newborns in this population tend to have higher proportional body fat as compared to their Caucasian counterparts [17]. Thus, this “Asian Indian phenotype” seems to have a genetic basis rather than an environmental and/or dietary explanation alone. 

The adipose tissue is dysregulated owing to obesity contributes to the genesis of diabetes by increasing the flux of free fatty acids (FFA), inflammatory cytokines, and various adipose tissue hormones [18,19,20,21]. Such adipose tissue dysfunction is characterized by an increase in the size of adipocyte (adipocyte hypertrophy) and the accumulation of pro-inflammatory macrophages [22,23]. Several studies done previously in Asian Indians found an association between visceral adipose tissue mass, qualitative changes in adipose tissue and altered adipocytokine flux, and diabetes along with metabolic syndrome and atherosclerosis [24,25,26]. For example, an association between visceral adipose tissue-related parameters such as its tissue mass, Visfatin [27,28], hsCRP [29], TNF-α [30,31], oxidative stress [32], and adipocyte size [33] with diabetes has been demonstrated. We previously showed qualitative molecular changes in visceral adipose tissue in Asian Indians by genome-wide transcriptome analysis [34]. Collectively, all these findings suggest that, in Asian Indians, visceral obesity is associated with pathological alteration in this adipose depot, which plays a significant role in the pathogenesis of diabetes mellitus and metabolic syndrome.

Although subcutaneous adipose tissue is usually considered protective, there exists another school of thought that believes it is responsible for diabetes and metabolic syndrome [35,36,37]. Some studies have shown that subcutaneous abdominal fat may play a vital role in the pathogenesis of both diabetes and metabolic syndrome [35]. In a few studies, subcutaneous fat is an even more significant determinant of insulin sensitivity [36]. As far as the Asian Indians are concerned, few studies point towards a potential role of subcutaneous abdominal adipose tissue in the pathogenesis of insulin resistance and possibly diabetes [37], but more studies are lacking. Recently we revealed the association between transcription profiles of peripheral subcutaneous adipose tissue and diabetes-related intermediate phenotypic traits of diabetes [38]. Whether this fat depot plays a causative or protective role in the pathogenesis of diabetes was not clarified by the study. 

Studies of Adipose tissue gene expression can potentially unravel the reason for linkage between adiposity and diabetes. Such studies can provide a more in-depth insight into the regulation of adipocytokines, which might be relevant to the inception of type 2 diabetes mellitus (T2D.) The study of adipose tissue gene expression could also perhaps pave the way for solving the mystery of which depot is more pathogenic, abdominal subcutaneous, or abdominal visceral adipose tissue. Gene expression profile studies in the adipose tissue of Asian Indians have been very rare. A recently published research on gene expression profiling in Asian Indians [39] showed reduced expression of uncoupling protein 2 (UCP2)in obese and diabetic patients, and this expression was further reduced in omental adipose tissue as compared to subcutaneous adipose tissue. Studies have also found [24] an increased expression of col6a3 and enhanced macrophage infiltration in adipose tissue along with increased systemic insulin resistance independent of body fat content in young Asian Indians. This finding suggests that adipose tissue dysfunction is associated with systemic insulin resistance regardless of adipose tissue mass. In a prior study [40], aimed at unraveling genome to phenome correlation in T2D, we first generated a physical and genetic interaction network of genes identified by Genome Wide Association Study (GWAS). After that, we created genome-wide expression profiles of multiple insulin-responsive tissues, including abdominal visceral and subcutaneous adipose tissue from non-diabetic and diabetic patients. Remarkably, the differentially expressed genes showed a significant overlap with the network genes. At the same time, the intersection showed enrichment of insulin signaling and other pathways consistent with T2D pathophysiology. In other words, this study suggests the role of T2D GWAS genes in adipose tissue dysfunction in diabetics. 

Despite all the evidence suggesting the role of abdominal adipose tissue in the pathogenesis of diabetes in Asian Indians, there are still several unresolved questions: Is there a difference in adipose tissue gene expression of visceral vs. subcutaneous region? How is the molecular alteration different in visceral vs. subcutaneous adipose tissue in diabetics? What is the relative role of visceral vs. subcutaneous adipose tissue in the pathogenesis of insulin resistance and diabetes?

To answer these as yet unanswered questions, in this study, we generated genome-wide gene expression profiles of visceral and subcutaneous depots of normal and diabetic individuals and determined various diabetes-related intermediate phenotypic traits: different anthropometric characteristics, measures of insulin resistance and secretion, glycemic control parameters, circulatory adipocytokine levels, adipocyte size, body fat distribution, etc. The generated data were analyzed for the generation of statistical and functional pieces of evidence of a correlation between gene expression profiles of these adipose depots and identification of the modules of co-expressed genes in these adipose depots showing significant correlation with the measured intermediate traits of T2D.

## 2. Materials and Methods

Fifty-six individuals comprising 33 non-diabetic controls and 23 diabetics (T2D) undergoing abdominal surgery were recruited for this study after obtaining their written consent. The institutional ethics committee of SMS Medical College (Number: 241MC/EC/2011), Jaipur, and the Indian Council of Medical Research (F.N. 5/4/8/2012-RMC) (ICMR), New Delhi, approved the study. We performed genome-wide transcription profiling in 16 non-diabetic controls and 19 T2D subjects, two samples from each subject—one from subcutaneous and one from visceral adipose tissue. The rest of the initially enrolled patients were excluded from transcription profiling because either their adipose tissue biopsy could not be obtained, or the sample did not match RNA quality requirements for microarray study. All methods were performed as per the relevant guidelines and regulations. The inclusion criteria for participants were: nonobese and type-2 diabetics (BMI < 30; age > 50) diagnosed as per American Diabetes Association (ADA) (2012) standards. The exclusion criteria were: the presence of infection, malignancy, and drugs affecting body fat/insulin resistance or adipocytokine expression such as thiazolidinediones, metformin, and glucocorticoids.

### 2.1. Clinical & Biochemical Assessment

Standard methods were used to obtain the anthropometric measurements, including body weight, height, waist-to-hip (W:H) ratio, and BMI. Supine blood pressure was measured using mercury sphygmomanometer (BPMR-120 Diamond delux, Industrial electronic and allied products, Maharashtra, India) after 10 min of rest. Blood samples were obtained at 8:00 a.m. next morning following an overnight fast of at least 8 h. Various biochemical parameters (e.g., serum glucose, lipid profile, triglycerides, low-density lipoprotein cholesterol (LDLc), high-density lipoprotein cholesterols (HDLc), and very-low-density lipoprotein cholesterol (VLDLc)) were measured on Kopran AU/400 (Olympus corporation, Shinjuku, Tokyo, Japan) fully automated analyzer. Serum insulin was measured using a chemiluminescent immunometric assay (Immulite 2000 machine, Siemens Healthineers AG, Erlangen, Germany). HbA1c was measured by turbidimetry method using BioSystems (Biosystems, S.A., Barcelona, Spain) kits. HOMA-IR and HOMA-B calculated insulin resistance was used to measure beta-cell function. Non-esterified fatty acids (NEFA, Randox Laboratories Ltd., Crumlin, UK), high sensitivity C reactive protein (hsCRP, Diagnostics Biocheme Canada Inc., Ontario, Canada), leptin (Lab systems Diagnostic Oy, Vantaa, Finland), and adiponectin (Diagnostics Biocheme Canada Inc., Ontario, Canada) were quantified using enzyme linked immunosorbent assay (ELISA) kits. Body fat content and distribution of patients undergoing abdominal surgery were estimated by dual-energy X-ray absorptiometry (DEXA) using the Hologic Explorer model (S/N91395 make, Hologic Canada ULC, Mississauga, Ontario, Canada). Biopsies were taken in formalin for processing of blocks and slides using standard protocols. Using Motic Panthera Moticam 5 trinocular microscope (BA210LED) (Motic Incorporation Ltd., Hong Kong, China), a representative image was taken for each section at 40×. For this microscope at 40× objective 1 pixel equals 0.121 µm^2^. The area of adipocytes was measured using Adobe Photoshop CC (Adobe Inc., San Jose, CA, USA) image analysis tool in pixels, and then, using the conversion factor, it was converted to µm^2^.

### 2.2. Transcription Profiling

RNA from each abdominal biopsy sample was isolated using the Qiagen RNeasy Mini Kit (Cat No. 74104). The quantification of samples was done using a Microfluidic-based capillary electrophoresis system (Bio-Rad Experion, Bio-Rad Laboratories, Inc., Philadelphia, PA, USA). Total RNA was made to undergo reverse transcription to synthesize the first-strand cDNA. The cDNA, thus formed was then converted to a double-stranded cDNA template during second-strand cDNA synthesis. Biotinylated ribonucleotide was subsequently incorporated by in vitro transcription reaction and then purified by the bead-based purification method. The purified biotin-labeled-cRNA was then fragmented using a fragmentation buffer, and then the sample was hybridized onto Affymetrix GeneChipPrimeView (Affymetrix Inc., Santa Clara, CA, USA). The obtained CEL files per sample were subjected to normalization and non-specific filtering using Bioconductor packages affy and genefilter. As primeview annotation package primeview.db was not available at Bioconductor, annotations file “PrimeView.na36.annot” was downloaded from the Affymetrix website, and the package was created using AnnotationForge and Human.db0 packages. Raw CEL files and series matrix files have been submitted to NCBI Genomic Expression Omnibus database (GEO Accession # GSE78721).

### 2.3. Real-Time PCR

We performed qPCR (quantitative polymerase chain reaction) on selected genes to check the reliability of microarray analyses. The oligonucleotide primer sequences used were raised using a universal probe library from Roche and synthesized as per the appropriate melting temperature (Tm), GC%. A total of 2 μg of RNA was isolated from the abdominal adipose tissue biopsy using Qiagen RNeasy Mini Kit (Cat No. 74104) and quantified using the microfluidic-based capillary electrophoresis system (Bio-Rad Experion). cDNA synthesis was done using the QuantiNova Reverse Transcription Kit (Cat No. 205411) and real-time PCR was done using the QuantiNova Probe PCR Kit (Cat. No 208252). The oligonucleotide primer sequences used in the qPCR analysis are listed in Table 1 (Supplementary Materials, Sheet S1).

### 2.4. Bioinformatics Analysis

For weighted gene correlation network analysis (WGCNA) analysis, samples in visceral and subcutaneous gene expression datasets were matched with the corresponding intermediate traits (Supplementary Materials, Sheet S2). One-step consensus network construction and module detection were then used; as biological networks follow scale-free topology, we selected a soft threshold power *β* = 15, which ensured that constructed network would be scale-free. Each module in this network comprises genes with similar expression profiles. The functional significance of relevant modules was then determined by pathway-based enrichment analysis against the human collection of Kyoto encyclopedia of genes and genomes (KEGG) pathways using WebGestalt [41]. 

To assess the level of similarity between both datasets at the statistical level, we used another component of meta-WGCNA [42], which could correlate measures of average gene expression, overall connectivity, and investigate module preservation between subcutaneous and visceral datasets. We also identified top meta-hub genes by determining genes with extremely high module membership (kME) values in both networks. 

Gene set enrichment analysis (GSEA) method ranks genes based on the correlation between their expression and the class label (such as diabetic and non-diabetics). It uses the Molecular Signatures Database (MSigDB) [43] as its knowledge base to calculate an enrichment score (ES) for the identification of overrepresented gene sets. We used a hallmark collection of fifty gene sets representing 48% of MSigDB gene sets. GSEA also provides functionality to extract the core members of high scoring gene sets—the leading-edge dubset that contributes to the ES. The leading-edge subset can be interpreted as the core of a gene set that accounts for the enrichment signal. We carried out separate GSEA of visceral and subcutaneous datasets and identified differentially regulated hallmark gene sets between normal and diabetic samples and between both depots of diabetics. We also extracted genes constituting leading-edge subsets in enriched gene-sets with False Discovery Rate (FDR)< 25% for the visceral dataset.

## 3. Results

### 3.1. Anthropometric and Biochemical Characteristics

The results of the anthropometric (mean and standard deviations) measures and its comparisons between non-diabetic controls and T2D patients are shown in Table 2. As the table illustrates, T2D patients and on-diabetic controls had similar anthropometric characteristics in terms of their age, height, weight, and BMI. The diabetics had higher waist circumference (*p* = 0.05) trend towards significance and greater waist-to-hip ratio (*p* = 0.02)

Table 3 demonstrates that, on comparing the biochemical parameters, there was no significant difference between the two groups in the levels of triglycerides, total cholesterol, very low density lipid cholesterol (VLDLc), low density lipid cholesterol (LDLc), high density lipid cholesterol (HDLc), creatinine, and HOMA β. Furthermore, the values of non-esterified fatty acids (NEFA), high-sensitivity C-reactive protein (hsCRP), leptin, adiponectin, and tumour necrosis factor α (TNFα) were also comparable in the two groups. 

However, the diabetics had significantly higher HOMA-R as compared to non-diabetics (*p* = 0.002). Diabetics also had significantly higher fasting insulin levels as compared to non-diabetics (*p* < 0.05). HbA1c levels were significantly higher in diabetics as compared to non-diabetics (*p* < 0.05). Interleukin-6 was significantly higher in diabetics as compared to non-diabetics (*p* = 0.02).

The comparison of the upper limb composition by DEXA analysis showed no significant difference in all parameters in the left and the right arm (Table 4). 

A comparison of the trunk fat content between the diabetics and the non-diabetics shows that the diabetics had significantly increased fat (*p* = 0.03). However, the difference in the percentage of fat content and the lean mass was non-significant between the two groups (Table 5).

A comparison of the composition of the lower limbs on DEXA shows no significant difference between the two groups (Table 6).

A comparison of the body composition characteristics for the whole body-head showed no significant difference in the lean body mass, the fat content, BMC, and the body fat percentage were also not statistically significant. However, there was a trend towards higher overall adiposity measured as total body fat (*p* = 0.06) (Table 7).

As shown in Table 8, on magnetic resonance imaging (MRI) imaging, the diabetics had considerably higher visceral adipose tissue mass as compared to the non-diabetics (*p* = 0.02). However, the differences in the subcutaneous and liver fat masses between these two groups were statistically not significant, but there was a trend towards the excess liver fat mass.

It is worth mentioning here that, in diabetics, despite similar molecular alterations in VAT and SAT, the adipose tissue mass was increased in the former only. This paradox might be since there are several determinants of adipose tissue mass such as adipocyte size (increased in visceral depot seen in this study; Table 9). Other factors explored in this study, such as the recruitment of mesenchymal cells towards adipocyte lineage, might also contribute to the increased VAT mass.

The comparison of adipocyte size between diabetics and non-diabetics shows that diabetics have greater visceral adipocyte size compared to non-diabetic controls with a significant *p*-value of 0.02 (Table 9). This finding suggests that higher visceral adipose tissue mass in diabetics could partly be due to adipocyte hypertrophy. 

Although the difference between the subcutaneous adipocyte size was not significant in the two groups, a trend towards larger subcutaneous adipocyte size was observed in diabetics. The greater adipocyte size is an indicator of adipocyte hypertrophy and poor adipogenesis, a hallmark of pathological adipose tissue. Therefore, these findings suggest that diabetes is associated with pathological adipose tissue in visceral and possibly in subcutaneous adipose tissue depot as well. 

Further, we compared the adipocyte cell size in the two compartments, i.e., VAT and SAT, in diabetics and the non-diabetic controls (intra-group comparison) (Table 10). We found no discernible difference between the two depots in terms of cell size within each group. This suggests that adipose tissue pathology measured in terms of adipocyte hypertrophy as adipogenesis defect is comparable in the subcutaneous and visceral adipose tissue depots within each group.

### 3.2. WGCNA of Visceral Fat 

**Figure 1:**
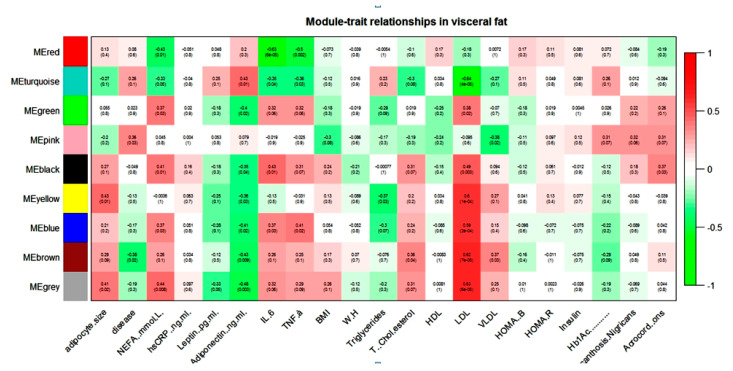
Module–trait relationship in visceral gene expression dataset.

WGCNA of the visceral dataset generated eight modules of co-expression genes, shown as turquoise, blue, brown, yellow, green, red, black, and pink in Figure 1. 

NEFA, IL-6, TNF-α, and LDL showed a positive correlation with the blue module and a negative correlation with the turquoise module in the visceral dataset. Adiponectin and NEFA showed a correlation with the maximum numbers of modules. Adiponectin showed a negative correlation with blue, brown, yellow, green, and black modules and a positive correlation with only the turquoise module in the visceral dataset. In contrast, NEFA showed a positive correlation with blue, green, and black modules and a negative correlation with turquoise and red modules. The brown module seems to contain genes that might play an important role in diabetic dyslipidemia because it showed a significant correlation with lipid parameters such as total cholesterol, LDL, VLDL, and diabetes phenotype. It is interesting note that most of these modules were either pathogenic or protective. For example, if any module showed a positive association with any pathogenic intermediate phenotypic trait (e.g., FFA), then the same module showed a negative correlation with the protective trait (e.g., adiponectin). 

WebGestalt-based functional analysis in blue, brown, and yellow enriched various KEGG pathways relevant to visceral adipose tissue biology is shown in Table 11.

The blue module in visceral datasets enriched various infection- and cancer-related pathways: *Acute myeloid leukemia, Salmonella infection, Amoebiasis, Proteoglycans in cancer,* and *Prion diseases*, point towards the disturbed immune status of visceral fat in diabetes. Adipose tissue pathobiology-related pathways, namely *HIF-1 signaling pathway, AGE-RAGE signaling pathway in diabetic complications, Focal adhesion,* and *Regulation of lipolysis in adipocytes*, were enriched. 

A transcription factor mediates the role of the HIF-1 signaling pathway in adipose physiology, hypoxia-inducible factor (HIF), high expression of which has been found in obesity-induced hypoxic conditions and may be the causative factor of adipose tissue inflammation. Pharmacological intervention of this pathway may, therefore, have the potential to enhance adipose functions [44].

The role of the *AGE-RAGE signaling pathway in diabetic complications* in the process of adipogenesis has been reported; over-expression of receptor for advanced glycation end products (RAGE) is also known to cause adipocyte hypertrophy and suppression of insulin-mediated glucose uptake in cultured 3T3-L1 cells [45].

The role of *Focal adhesion* in adipocyte differentiation has already been established. One study has revealed that focal adhesion kinases (FAK) participate in adipocyte differentiation. Their cleavage by calpain is required to fulfill the final maturation of adipocytes [46].

*Regulation of lipolysis in adipocytes* is also relevant in T2D pathology. Lipolysis in adipocytes is mediated by adipose triglyceride lipase (ATGL), hormone-sensitive lipase (HSL), and mono-glyceride lipase. Hormonal and nutritional factors tightly regulate the activity of the lipolytic pathway. However, the failure to efficiently suppress lipolysis when FFA demands are low can have serious metabolic consequences and might be a key mechanism in the development of type 2 diabetes in obesity [47].

The majority of pathways in the brown module (visceral dataset) were related to housekeeping activities such as protein synthesis, cell division, and metabolism. *MicroRNAs in cancer* might be relevant to visceral fat physiology. Several recent studies have reported that numerous miRNAs influence adipogenic differentiation of mesenchymal stem cells (MSCs), and there is a potential for microRNA-based therapy for treating metabolic disorders by adipogenesis regulation. Elevated levels of miR-143 were detected in differentiated white adipocytes, and its inhibition resulted in reduced adipocyte differentiation. Furthermore, the ERK1/2 pathway negatively regulates adipocyte differentiation, and it has been reported that this reduction in adipogenesis is mediated through miR-375 [48].

Enrichment of the *Hippo signaling pathway* in the yellow module (visceral datasets) may be relevant to adipose tissue biology. It has been reported that Lats2—one of the core kinases of the Hippo pathway—inhibits the proliferation of 3T3-L1 cells but promotes their differentiation through the PPARγ-mediated transcription program [49]. We believe that pharmacological inhibition of this pathway may provide a valuable opportunity for diabetes management. Furthermore, *Pathways in cancer* are also linked to obesity; it is now being accepted that dysregulation of adipocyte function and obesity-driven chronic inflammation are the main culprits in adiposity-induced tumorigenesis [50]. This relationship is particularly evident in cancers that grow in adipocyte-rich environments such as breast carcinomas or cancers that have a propensity to metastasize to fat-rich sites, such as ovarian or gastric malignancies. In addition to acting as a local paracrine signaling molecule, adipokines also exert systemic effects and allow for communication with distant sites. The increased levels of adipose tissue-derived factors, such as TNF-α, IL-6, IL-8, macrophage chemoattractant protein (MCP-1), and leptin, and their role in tumor progression have been well-documented [51].

### 3.3. WGCNA of Subcutaneous Fat 

**Figure 2:**
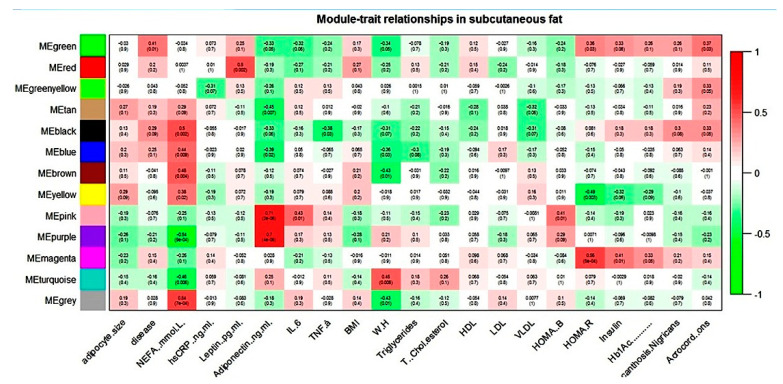
Module–trait relationship in subcutaneous gene expression dataset.

WGCNA of the subcutaneous dataset generated twelve modules of co-expression genes, shown as turquoise, blue, brown, yellow, green, red, black, pink, magenta, purple, green-yellow, and tan in Figure 2. 

NEFA showed correlation with six modules and adiponectin with four modules in the subcutaneous dataset. For all these modules, NEFA and adiponectin showed an association that was always reverse in direction. The magenta module (subcutaneous dataset) seems biologically relevant to T2D pathology, as it correlated with all the three hallmark parameters of the disease: HOMA-R, insulin, and Hb1Ac.

WebGestalt-based functional analysis of various blue, brown, and magenta (subcutaneous dataset) enriched KEGG pathways relevant to adipose tissue biology is shown in Table 12.

All pathways in the blue module (subcutaneous dataset) were related to anabolic processes such as biosynthesis of amino acids, sugar, and protein synthesis. In the brown module of the subcutaneous dataset, we found enrichment of relevant pathways: *Regulation of lipolysis in adipocytes, PPAR signaling pathway, Insulin signaling pathway,* and *AMPK signaling pathway.* Activation of *the AMPK signaling pathway* acts as a sensor for cellular energy status. Its activation leads to the inhibition of pre-adipocyte differentiation and adipogenesis through increased phosphorylation of its substrate, acetyl-CoA carboxylase (ACC) [52]. Interestingly a widely prescribed drug for type 2 diabetes, metformin, inhibits hepatic gluconeogenic program by activation of AMPK pathway [53]; long-term usage of it may, therefore, have negative consequences on normal adipose physiology.

Adipogenesis is regulated by complex and highly regulated gene expression that includes *Signaling by peroxisome proliferator-activated receptor γ (PPARγ)*, which induces differentiation of pre-adipocytes to adipocytes and is essential for this process. It has been reported that PPARγ mRNA and protein levels are downregulated by fasting and insulin-deficient diabetes. At the same time, a diet rich in fatty acids increases adipose tissue expression of PPARγ in normal mice and induces PPARγ2 expression in the liver of obese mice [54].

The *ECM-receptor interaction* is another critical pathway, deregulation in which may predispose towards adiposopathy. In adipose tissues, mature adipocytes and their progenitor cells (pre-adipocytes) exist within a three-dimensional (3D) network of ECM proteins. Adipose tissue function is regulated by the physiological interaction between cells and a variety of ECM proteins. The collagen family is the largest group of ECM proteins. The excess collagen deposition in adipose tissues was observed along with inflammatory tissue damage, which is characterized by the infiltration of neutrophils, lymphocytes, and macrophages. Thus, fibrotic tissue damage is perceived by many as a process secondary to tissue inflammation, whose pathological impact on obesity and metabolism has been extensively studied in recent years [55].

In the magenta module, the majority of enriched pathways were found to be related to immune- and inflammatory processes. 

### 3.4. WGCNA for Statistical Comparison of Visceral and Subcutaneous Depots

**Figure 3:**
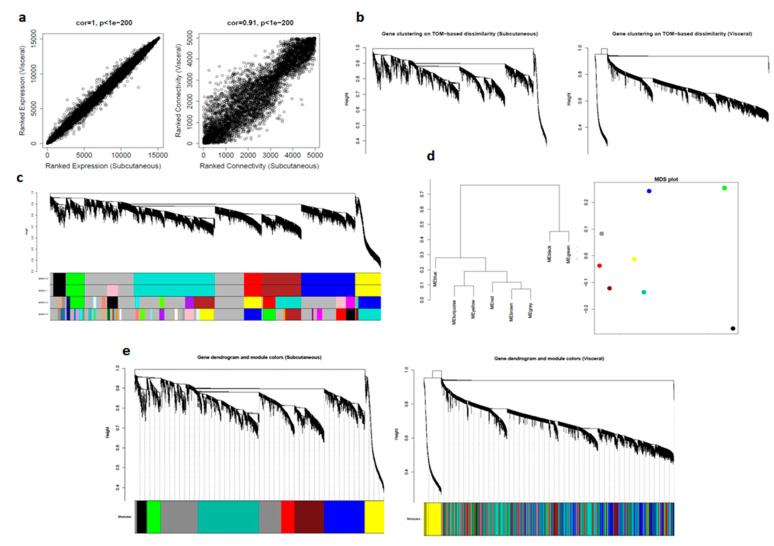
Comparison of visceral and subcutaneous datasets using WGCNA: (**a**) correlation between datasets in average gene expression, and overall connectivity; (**b**) gene dendrograms on TOM-based dissimilarity; (**c**) module choices for deep split values; (**d**) module eigengene visualization; and (**e**) preservation of modules in datasets.

We first compared gene expression profiles of subcutaneous and visceral adipose tissues by correlating their measures of average gene expression and overall connectivity. The correlations were positive, and the *p*-values were significant in both cases. This significant p-value suggested that the datasets are comparable. The correlations and *p*-values were better for expression than for connectivity (Figure 3a).

For computational reasons and simplicity, we selected the top 5000 most expressed genes in the subcutaneous dataset and then calculated values for adjacency, disTOM, and geneTree for these genes in both the datasets. Since there were many distinct branches in both the network, we can assume this to be useful data (Figure 3b).

Next, we considered subcutaneous dataset as control and determined modules for four deep split values (0–3) and selected dpSplit = 0, which was also consistent with deSplit = 1 (Figure 3c).

For further quantitative assessment, we visualized the dendrogram of the module eigengenes and the multidimensional scaling (MDS) plot of the module eigengene, where the x-axis was the first PC and the y-axis was the second PC: modules that grouped have a relatively similar expression (Figure 3d).

To qualitatively assess how well our modules in the subcutaneous network were preserved in the visceral network, we imposed the modules from subcutaneous datasets on the network for visceral dataset, and then plotted the resulting networks. We could see from the fact that these module labels still grouped in visceral (right) that there is excellent preservation. It is important to note that it is often not possible to see a distinct grouping of model labels in the second dataset, even if there is significant module preservation (Figure 3e).

To quantify this result, we took advantage of another function built into the WGCNA library: *module preservation*. This function assesses how well a module in one study is preserved in another study using several strategies and outputs a single Z-score summary (Table 13).

In general, the higher is the value of “Zsummary.pres,” the more preserved is the module between datasets: 5 < Z < 10 indicates moderate preservation, while Z > 10 indicates high preservation. The “grey” module contains an uncharacterized gene, while the “black” module contains random genes. In general, these modules should have lower Z-scores than most of the other modules. In this case, we found that all modules were very well preserved. We then obtained the module membership (kME) values, along with their associated *p*-values for subcutaneous and visceral datasets. We also identified the top 10 hub genes in the networks by determining genes with extremely high kME values in both networks (Table 14).

### 3.5. GSEA for Functional Comparison of Visceral and Subcutaneous Depots 

We identified differentially regulated MSigDB-hallmark pathways for normal vs. diabetics in both visceral and subcutaneous and between diabetics in the two depots using GSEA (Supplementay Materials, Sheet S3). 

On comparison of the visceral depot, 42 gene sets were found to be upregulated in diabetics, of which 19 gene sets were significant at FDR < 25% including various immune system and inflammation-related pathways such as *Interferon α, γ, Allograft rejection, IL-6 medicated JAK-STAT signaling, Complement activation, TGF-β signaling,* and *Inflammation*.

GSEA analysis of subcutaneous tissue also reported various upregulated hallmark inflammatory gene sets such as IL-2, IL-6, and TNF-α signaling in diabetics. However, only one gene set (IL2-STAT5 signaling) was significant at FDR< 25%. Interestingly, 13 out of the 19 significant pathways enriched in visceral GSEA were also present among the top 20 pathways in the subcutaneous analysis. This finding corroborates our hypothesis that diabetes pathology inflicts similar molecular alternations in visceral and subcutaneous fat, but with more intense clinical presentation in visceral than in subcutaneous. 

GSEA program also provides functionality to conduct leading-edge analysis (LEA) to identify subsets of genes in enriched gene sets with the highest contribution in enrichment signals. LEA of visceral GSEA identified 30 genes involved in 4–7 gene sets: *IL6, IL1B, SERPINE1, CDKN1A, TIMP1, ID2, CD44, BMP2, IRF1, FAS, TGFB1, PLAUR, ATF3, OLR1, F3, TFPI2, CXCL10, CCL2, ICAM1, IL18, PLAT, CCND2, TNFAIP3, MYC, BTG2, TAP1, GADD45A, TOP2A, TLR2,* and *CXCL1*. These sets of genes can be investigated for potential disease biomarkers and as possible anti-diabetic drug targets. 

To confirm our hypothesis, we analyzed gene expression differences among diabetics in both depots. None of the gene sets were found significant at FDR < 25%. Enriched gene sets appeared to be clinically non-suggestive (except pancreatic β-cells) and, therefore, may be the result of inherent microarray noise. 

## 4. Discussion

The major objectives of the study were to investigate whether visceral and subcutaneous adipose tissue depots show molecular pathological changes in diabetics, whether the pattern of these molecular changes is similar in the two adipose depots, and whether the transcription profiles of visceral and subcutaneous adipose tissue show any association with diabetes pathophysiology-related phenotypic traits.

Transcription profiling and adipocyte size estimation were used for adipose tissue molecular pathology assessment. Correlation study of the transcription profiles with diabetes-related intermediate phenotypic traits was done to assess their role in the diabetes pathophysiology. The results of this study suggest that: (i) Diabetes in Asian Indians is associated with pathologic alterations of both visceral and subcutaneous adipose tissue characterized by adipocyte hypertrophy and altered expression of genes that broadly enrich inflammation- and adipogenesis-related pathways. (ii) The transcriptional changes in both visceral and subcutaneous adipose tissue are qualitatively similar, although more intense in the former. (iii) Modules of co-expressed genes in both these adipose depots show pathogenic as well as protective association with diabetes-related intermediate phenotypic traits. Therefore, these findings suggest that both adipose depots show pathological changes and both possibly play a role in the pathophysiology of diabetes. Whether their role is protective or pathogenic depends on the modules of the co-expressed genes they contain rather than their location (visceral or subcutaneous) alone. In other words, these findings do not support the hypothesis that visceral adipose tissue is pathogenic and subcutaneous adipose tissue is protective in the pathophysiology of diabetes in this population. 

In Asian Indians, several clinical and epidemiological studies have shown an association between the visceral adipose tissue mass (but not the subcutaneous adipose tissue mass) and the incidence of diabetes and metabolic syndrome [56]. Such findings suggest that these two fat depots are biologically different organs with different molecular functions and possibly of different embryological origins. To test this hypothesis, in this study, we compared the transcription profiles in these adipose depots. On WGCNA analysis for statistical comparison, we observed that all the modules of co-expressed genes representing the top 5000 expressed genes in subcutaneous adipose tissue were preserved in visceral adipose tissue. However, four out of nine modules showed only moderate preservation, suggesting that qualitative similarity in the transcription profiles of visceral and subcutaneous adipose tissue in Asian Indians was associated with minor quantitative differences. In addition, on functional comparison of both adipose depots in diabetics, none of the gene sets were found significant at FDR <25%. Moreover, these non-significantly enriched gene sets appeared to be clinically non-suggestive (except pancreatic β-cells) and, therefore, may be the result of inherent microarray noise. This evidence suggests that, even if there are minor quantitative differences in transcription profiles of visceral and subcutaneous adipose tissue among diabetics, they do not have any functional significance. Interestingly, 13 out of 19 significant gene sets enriched in visceral adipose tissue in diabetics as compared to controls were also present among top 20 pathways on a similar analysis of the subcutaneous adipose tissue, thus supporting the fact that, at the molecular level, diabetes is associated with qualitatively similar molecular alterations in both visceral and subcutaneous adipose tissues. 

Several previous studies [57,58,59,60,61] compared visceral versus adipose tissue gene expression profiles. In these studies, the quantitative analysis of differential expressed genes revealed several differentially expressed genes (DEGs) such as complement pathways, fatty acid synthase, adiponectin, gastric inhibitory peptide, etc. These findings are inconsistent with those of the present study. In our study, we found a minor quantitative difference in the transcription profiles between the visceral and subcutaneous adipose tissue, although qualitatively they were similar in diabetics. We also compared the adipose tissue transcription profiles of diabetics with those of the controls, separately for visceral and subcutaneous compartments. Several immune systems-related genes showed altered expression among diabetics in both adipose depots. However, at the significance level of FDR <25%, 19 out of 42 gene sets in visceral and none in the subcutaneous compartment showed altered expression in diabetics. Keeping in view the fact that diabetics had larger adipocyte size (although statistically insignificant in subcutaneous adipose depot), the modules of co-expressed genes in both depots showed association with its intermediate phenotypic traits and these modules of co-expressed genes enriched inflammation- and adipogenesis-related pathways; all such findings suggest that diabetes is associated with pathologic adipose tissue in both compartments. In two recent studies [38,62], we showed that diabetes is associated with similar pathologic changes in peripheral subcutaneous adipose tissue, and these pathologic changes show an association with diabetes-related intermediate phenotypic traits. Taken together, this evidence explains that the “thin fat” phenotype of Asian Indians is due to qualitative pathologic alterations in the adipose tissue. In the case of the abdomen, these changes are more intense in the visceral compartment. In most previous studies, Asian Indians, as compared to other ethnicities, have shown larger visceral adipose tissue mass on radiological imaging and even more so in diabetics as compared to controls [17,63,64]. The MRI results of the present study also report the same. However, this quantitative trait among diabetics is probably due to the qualitative pathologic process of poor adipogenesis in diabetics because adipocytes in them showed hypertrophy measured as large adipocyte size. 

Another important finding of this study is the identification of modules of co-expressed genes showing association with diabetes pathophysiology-related intermediate phenotypic traits. Interestingly, most of these traits were either pathogenic or protective. A pathogenic trait means showing a positive association with the pathogenic trait (i.e., FFA, adipocyte size, etc.) and a negative association with protective traits (i.e., adiponectin). In protective traits, this relationship was reversed. It is proposed here that these modules can potentially serve as “units” of adipose tissue factor in the pathogenesis of dysmetabolism in diabetics, instead of specific depots such as visceral, subcutaneous, etc. A specific depot might vary in its pathogenicity depending on the content of different types of modules and their quantity. These modules of co-expressed genes not only show orchestrated expression and function but are also likely to share a common regulatory mechanism. Therefore, future diagnostic and therapeutic strategies aimed at treating diabetes-associated adipose tissue dysfunction and insulin resistance might be targeted towards these modules. However, what we have proposed is just the beginning of the concept. 

## 5. Conclusions

Diabetes is associated with adipose tissue pathology in both subcutaneous and visceral depots. This adipose tissue pathology is suggested by adipocyte hypertrophy and altered gene expressions enriching the inflammation- and adipogenesis-related pathways seen in our study. Qualitatively, this pathology is similar in both the visceral and subcutaneous compartments, with the changes being more intense in the former. The modules of co-expressed genes in both compartments show pathogenic as well as protective association with diabetes-related intermediate phenotypic traits. Our findings, therefore, suggest that both adipose tissue compartments undergo molecular alterations and the effect of these alterations depends upon the modules of the co-expressed gene rather than their location alone.

## 6. Limitations of the Study

The sample size taken for the study is small and requires further validation research incorporating a larger sample size and the latest technological advancements, e.g., RNA sequencing, etc.

## Figures and Tables

**Table 1:** Primer pairs used in qPCR.

S. No.	Gene	Primer Pairs
1	*PIK3R1*	Forward, 5′- CTCTGGTTGGTGTGGGCT-3′and Reverse, 5′- AGGAAGAGAGTCGCGGCA-3′
2	*IL7R*	Forward, 5′- GGAAAATGTCATGCTCCTGG -3′ and Reverse, 5′- CATAAAATCTGTATGACCTGCCC -3′
3	*KRAS*	Forward, 5′- GTACGCCCGTCTGAAGAAGA -3′and Reverse, 5′- CCCTAATTCATTCACTCGCC -3′
4	*USP8*	Forward, 5′- GCGGTGGAAGAGAGAGGAGT -3′and Reverse, 5′- AGACTCAAGGTTGGGCCTTT -3′
5	*PPARA*	Forward, 5′- GAGGACACACACCGAGGACT -3′and Reverse, 5′- GCAGCTGGAGGAACAAACAC -3′
6	*TAT.*	Forward, 5′- GCTGCTGAGTTGTCATTCCA -3′ and Reverse, 5′- GCTCAATCAGTCACCACTGC -3′
7	*AKT2*	Forward, 5′- AGGCGCTGTTGTTATGCTCT -3′and Reverse, 5′- GGTCTGATAAGATGCGGTGG -3′
8	*LIPE*	Forward, 5′- AGGCCTAAATTGGGATGCTT -3′and Reverse, 5′- GAGTCTTCGATTCTGGCTGG -3′
9	*AGPAT2*	Forward, 5′- CACCTAGCCCTTCCCTGC -3′ and Reverse, 5′- GGGAAGCCCAGAAGAAAGTT -3′
10	*LEP.*	Forward, 5′- GATCGGGCCGCTATAAGAG -3′and Reverse, 5′- GTCCAGAACTAAGCCATCCG -3′
11	*LMNA*	Forward, 5′- CATGCCGGGAGTTGTAGTTT -3′and Reverse, 5′- TTCATACCCGCTCTGTTTCC -3′
12	*FOS.*	Forward, 5′- TCTGAGACAGGAACTGCGAA -3′and Reverse, 5′- CTCATCTACTGGAGCGTCCC -3′
13	*SAT1*	Forward, 5′- GAGAGGTCCCACCTCACG -3′and Reverse, 5′- AGCTCAGGGGAACGGAAT -3′
14	*ACTB*	Forward, 5′- CCAACCGCGAGAAGATGA -3′and Reverse, 5′- CCAGAGGCGTACAGGGATAG -3′
15	*G6PD*	Forward, 5′- AACAGAGTGAGCCCTTCTTCA -3′and Reverse, 5′- GGAGGCTGCATCATCGTACT -3′

**Table 2:** Baseline characteristics of the T2D patients and non-diabetic controls.

Characteristic	T2D Patients	Non-Diabetic Controls	*p*-Value
Age (years)	51.96 ± 11.93	54.09 ± 9.21	0.46
Height (Meters)	1.62 ± 0.08	1.59 ± 0.07	0.12
Weight (kgs)	64.29 ± 12.11	62.38 ± 17.75	0.64
BMI (kg/m^2^)	24.45 ± 4.97	24.66 ± 7.00	0.90
Waist circumference (cm)	96.74 ± 8.28	90.4 ± 13.88	0.05
Waist-to-hip ratio	1.00 ± 0.07	0.93 ± 0.05	0.02

**Table 3:** Biochemical parameters between the two groups.

Biochemical Parameters	T2D Patients	Non-Diabetic Controls	*p*-Value
Triglyceride (mg/dL)	169.58 ± 66.35	178.24 ± 121.73	0.76
Total cholesterol (mg/dL)	182.74 ± 51.15	195.17 ± 36.86	0.38
HDL (mg/dL)	39.68 ± 7.84	41.38 ± 4.63	0.41
LDL (mg/dL)	97.56 ± 32.50	108.77 ± 17.88	0.20
VLDL (mg/dL)	32.17 ± 16.81	44.77 ± 27.05	0.06
S. Creatinine (mg/dL)	0.90 ± 0.28	0.89 ± 0.18	0.92
HOMA-β	124.12 ± 80.08	157.72 ± 82.89	0.14
HOMA- R	13.36 ± 12.09	1.91 ± 1.36	<0.001
Insulin (mU/L)	28.31 ± 15.37	9.01 ± 5.64	<0.001
HbA1C (%)	7.77 ± 1.59	5.57 ± 0.78	<0.001
NEFA (mmol/L)	0.76 ± 0.38	0.69 ± 0.33	0.59
hsCRP (ng/mL)	9367.68 ± 6737.16	6902.84 ± 4645.43	0.23
Leptin (ng/mL)	86.27 ± 196.78	59.8 ± 157.26	0.66
Adiponectin (ng/mL)	51.27 ± 46.10	98.35 ± 153.69	0.26
Interleukin-6 (IL-6) (pg/mL)	60.90 ± 75.04	23.23 ± 27.43	0.02
TNF-alpha (pg/mL)	90.84 ± 236.74	26.11 ± 33.6	0.15

**Table 4:** Comparison of body composition on DEXA of upper limbs.

Parameter	T2D Patients	Non-Diabetic Controls	*p*-Value
**Left arm**			
Bone mineral Content (BMC) (g)	125.73 ± 44.15	140.32 ± 92.49	0.67
Fat (g)	1632.91 ± 1336.60	1595.62 ± 1036.04	0.94
Lean (g)	1986.26 ± 830.36	2382.19 ± 1858.56	0.56
% Fat	39.35 ± 13.29	40.32 ± 8.64	0.83
**Right arm**			
BMC (g)	133.70 ± 45.14	116.11 ± 40.58	0.34
Fat (g)	1804.08 ± 1723.42	1501.01 ± 826.08	0.57
Lean (g)	2008.02 ± 746.26	2383.37 ± 1470.10	0.49
% Fat	40.69 ± 14.28	38.39 ± 8.21	0.62

**Table 5:** Comparison of Trunk composition by DEXA.

Parameter	T2D Patients	Non-Diabetic Controls	*p*-Value
Fat (g)	10543.02 ± 4092.59	7446.59 ± 3689.08	0.03
Lean (g)	20811.17 ± 3321.58	18416.49 ± 5362.18	0.19
% Fat	32.72 ± 7.1	29.61 ± 7.32	0.26

**Table 6:** Comparison between lower limb body composition between T2D patients and Non-diabetic controls.

Parameter	T2D Patients	Non-Diabetic Controls	*p*-Value
**Left Leg**			
BMC (g)	378.50 ± 96.87	302.88 ± 110.13	0.06
Fat (g)	4023.98 ± 1992.90	3015.75 ± 1439.28	0.11
Lean (g)	6638.05 ± 1483.48	5442.44 ± 1873.61	0.06
% Fat	35.09 ± 9.72	35.21 ± 7.82	0.97
**Right Leg**			
BMC (g)	383.09 ± 91.80	319.36 ± 148.82	0.18
Fat (g)	4087.24 ± 2142.90	3297.09 ± 1379.53	0.23
Lean (g)	6681.75 ± 1611.29	5614.40 ± 2234.53	0.15
% Fat	35.26 ± 10.04	35.63 ± 7.25	0.91

**Table 7:** Comparison of whole-body composition on DEXA (body-head).

Parameter	T2D Patients	Non-Diabetic Controls	*p*-Value
BMC(g)	1453.95 ±3 43.96	1205.05 ± 475.98	0.12
Fat(g)	21,689.16 ± 9331.46	16,245.56 ± 6011.51	0.06
Lean (g)	37,197.17 ± 6165.82	31,776.79 ± 8659.18	0.06
% Fat	34.79 ± 8.62	32.55 ± 7.02	0.43

**Table 8:** Abdominal fat comparison between the diabetics and non-diabetics on MRI.

Characteristic	Diabetics	Non-Diabetics	*p*-Value
Visceral Fat (cm^2)^)	142.42 ± 93.91	78.72 ± 33.72	0.02
Subcutaneous fat (cm^2^)	138.20 ± 96.83	135.73 ± 74.34	0.93
Liver fat (% fat signal intensity)	10.27 ± 7.37	7.76 ± 4.05	0.28

**Table 9:** Comparison of the adipocyte cell size between diabetics and non-diabetics.

Adipose Tissue Site	Diabetics	Non-Diabetics	*p*-Value
Visceral fat (pixels)	172,208.7 ± 47,740.9	134,851.55 ± 46,097.86	0.02
Visceral fat (µm^2^)	20,837.25 ± 5776.65	16,317.04 ± 5577.84	0.02
Subcutaneous fat (pixels)	167,892.62 ± 82,111.68	135,429.65 ± 58,614.09	0.20
Subcutaneous fat (µm^2^)	20,315.01 ± 9935.51	16,386.99 ± 7092.31	0.20

**Table 10:** Comparison of visceral and subcutaneous adipocyte cell size in diabetics and non-diabetics.

Adipose Tissue Site	Visceral Fat	Subcutaneous Fat	*p*-Value
Diabetics (pixels)	172,208.7 ± 47740.9	167,892.62 ± 82,111.68	0.82
Diabetics (µm^2^)	20,837.25 ± 5776.65	20,315.01 ± 9935.51	0.82
Non-diabetics (pixels)	134,851.55 ± 46,097.86	135,429.65 ± 58,614.09	0.96
Non-diabetics (µm^2^)	16,317.04 ± 5577.84	16,386.99 ± 7092.31	0.96

**Table 11:** Enriched KEGG pathways in relevant modules of visceral datasets.

**Blue Modules**				
**S. No.**	**KEGG ID**	**Pathway**	**# Genes**	**FDR.**
1	hsa04142	Lysosome	33	0.000309
2	hsa04066	HIF-1 signaling pathway	29	0.000309
3	hsa04933	AGE-RAGE signaling pathway in diabetic complications	28	0.000426
4	hsa04510	Focal adhesion	45	0.000426
5	hsa04923	Regulation of lipolysis in adipocytes	18	0.00154
6	hsa05221	Acute myeloid leukemia	18	0.00168
7	hsa05132	Salmonella infection	22	0.00721
8	hsa05146	Amoebiasis	24	0.00819
9	hsa05205	Proteoglycans in cancer	40	0.00819
10	hsa05020	Prion diseases	12	0.00819
**Brown Modules**				
**S. No.**	**KEGG ID**	**Pathway**	**# Genes**	**FDR.**
1	hsa03040	Spliceosome	34	2.19 × 10^−8^
2	hsa04120	Ubiquitin mediated proteolysis	26	1 × 10^−3^
3	hsa04110	Cell cycle	21	2.78 × 10^−2^
4	hsa03008	Ribosome biogenesis in eukaryotes	15	5.82 × 10^−2^
5	hsa03013	RNA transport	24	5.82 × 10^−2^
6	hsa00970	Aminoacyl-tRNA biosynthesis	10	5.82 × 10^−2^
7	hsa04115	p53 signaling pathway	13	6.3 × 10^−2^
8	hsa05166	HTLV-I infection	31	1.82 × 10^−1^
9	hsa00020	Citrate cycle (TCA cycle)	7	1.82 × 10^−1^
10	hsa05206	MicroRNAs in cancer	19	4.32 × 10^−1^
**Yellow Modules**				
**S. No.**	**KEGG ID**	**Pathway**	**# Genes**	**FDR.**
1	hsa04610	Complement and coagulation cascades	9	1.08 × 10^−2^
2	hsa04015	Rap1 signaling pathway	13	7.06 × 10^−2^
3	hsa05205	Proteoglycans in cancer	11	2.52 × 10^−1^
4	hsa00410	beta-Alanine metabolism	4	2.52 × 10^−1^
5	hsa04390	Hippo signaling pathway	9	2.52 × 10^−1^
6	hsa00350	Tyrosine metabolism	4	2.52 × 10^−1^
7	hsa05143	African trypanosomiasis	4	2.52 × 10^−1^
8	hsa04512	ECM-receptor interaction	6	2.72 × 10^−1^
9	hsa05200	Pathways in cancer	16	2.87 × 10^−1^
10	hsa04550	Signaling pathways regulating pluripotency of stem cells	8	2.91 × 10^−1^

**Table 12:** Enriched KEGG pathways in relevant modules of subcutaneous datasets.

**Blue Modules**				
**S. No.**	**KEGG ID**	**Pathway**	**# Genes**	**FDR.**
1	hsa04210	Apoptosis	35	6.84 × 10^−3^
2	hsa04141	Protein processing in endoplasmic reticulum	39	6.84 × 10^−3^
3	hsa00051	Fructose and mannose metabolism	13	7.93 × 10^−3^
4	hsa01230	Biosynthesis of amino acids	21	1.57 × 10^−2^
5	hsa04142	Lysosome	28	4.9 × 10^−2^
6	hsa05221	Acute myeloid leukemia	16	4.9 × 10^−2^
7	hsa01200	Carbon metabolism	26	4.9 × 10^−2^
8	hsa00520	Amino sugar and nucleotide sugar metabolism	14	4.9 × 10^−2^
9	hsa04912	GnRH signaling pathway	22	4.9 × 10^−2^
10	hsa01522	Endocrine resistance	23	4.9 × 10^−2^
**Brown Modules**				
**S. No.**	**KEGG ID**	**Pathway**	**# Genes**	**FDR.**
1	hsa00620	Pyruvate metabolism	11	3.59 × 10^−6^
2	hsa00640	Propanoate metabolism	9	4.13 × 10^−5^
3	hsa01100	Metabolic pathways	62	2.7 × 10^−3^
4	hsa01200	Carbon metabolism	13	2.7 × 10^−3^
5	hsa04923	Regulation of lipolysis in adipocytes	8	1.28 × 10^−2^
6	hsa03320	PPAR signaling pathway	9	1.28 × 10^−2^
7	hsa04910	Insulin signaling pathway	13	1.28 × 10^−2^
8	hsa04152	AMPK signaling pathway	12	1.31 × 10^−2^
9	hsa00500	Starch and sucrose metabolism	6	2.18 × 10^−2^
10	hsa04512	ECM-receptor interaction	9	2.33 × 10^−2^
**Magenta Modules**				
**S. No.**	**KEGG ID**	**Pathway**	**# Genes**	**FDR.**
1	hsa04640	Hematopoietic cell lineage	6	5.68 × 10^−3^
2	hsa04060	Cytokine-cytokine receptor interaction	8	1.86 × 10^−2^
3	hsa04658	Th1 and Th2 cell differentiation	5	1.86 × 10^−2^
4	hsa04660	T cell receptor signaling pathway	5	2.26 × 10^−2^
5	hsa04659	Th17 cell differentiation	5	2.26 × 10^−2^
6	hsa04662	B cell receptor signaling pathway	4	4.21 × 10^−2^
7	hsa05340	Primary immunodeficiency	3	5.51 × 10^−2^
8	hsa04064	NF-kappa B signaling pathway	4	8.41 × 10^−2^
9	hsa04630	Jak-STAT signaling pathway	4	4.5 × 10^−1^
10	hsa04062	Chemokine signaling pathway	4	7.08 × 10^−1^

**Table 13:** Module preservation summary between visceral and subcutaneous datasets.

S. No.	Module	Module Size	Z Score for Preservation
1	Yellow	385	14.34
2	Blue	400	14.03
3	Red	264	13.97
4	Turquoise	400	13.74
5	Brown	400	11.39
6	Gold	100	8.95
7	Green	271	8.39
8	Grey	400	7.30
9	Black	191	6.90

**Table 14:** Top 10 meta-hub genes in preserved modules between visceral and subcutaneous datasets.

S. No.	Modules	Top 10 Genes with High kME
1	Black	*CD53, RNASET2, NAGK, CDC42SE1, FYB1, LCP1, RASSF2, LAPTM5, CYBA, CYTH1*
2	Blue	*SEC24B, PSMD10, UBE3A, USP33, STXBP3, IBTK, RAB28, TMCO1, DENR, NARS*
3	Brown	*PPP2CB, STRAP, RDX, RTN3, TACC2, PARVA, RHOQ, PHYH, PRDX6, ATL3*
4	Green	*SF3B1, ZNF655, SPG11, PHC3, NR2C2, ZRANB2, MIOS, POLR2B, CD47, HNRNPH1*
5	Grey	*NGRN, MCFD2, RDX, VPS26A, ATP2B4, DCTN4, UBE2E2, SMIM7, RRAGA, ATL3*
6	Red	*PTPRF, TMEM184B, VAPB, RRAGC, ETFB, TRIM8, COPRS, KCTD20, CYB5R1, TKT*
7	Turquoise	*CDC37, PYCR2, ENSA, U2AF2, UBE2L3, ARF1, CTBP1, GSK3A, WDR82, PSMB6*
8	Yellow	*KRTAP6-1, KRTAP10-1, PKMYT1, BLOC1S3, SHF, CRIP3, KCTD17, RARG, P.M.L., LINC01547*

## Supplementary Materials

The following are available online at https://www.mdpi.com/2218-273X/10/9/1230/s1.
